# Intracranial pressure trends and clinical outcomes after decompressive hemicraniectomy in malignant middle cerebral artery infarction

**DOI:** 10.1186/s13613-024-01412-0

**Published:** 2024-11-27

**Authors:** Jae Wook Jung, Ilmo Kang, Jin Park, Seungjoo Lee, Sang-Beom Jeon

**Affiliations:** 1 Department of Neurology, Asan Medical Center, University of Ulsan College of Medicine, 88, Olympic-ro 43-gil, Songpa-gu, Seoul, 05505 Korea; 2 Department of Neurosurgery, Asan Medical Center, University of Ulsan College of Medicine, Seoul, Korea

**Keywords:** Cerebral infarction, Decompressive hemicraniectomy, Intracranial pressure, Neurocritical care

## Abstract

**Background:**

Malignant middle cerebral artery infarction (MMI) is associated with a high incidence of severe disability and mortality. Decompressive hemicraniectomy has become a recognized treatment that can improve the prognosis for patients if performed within a certain time window. Nevertheless, despite this intervention, a mortality rate of approximately 20–40% persists following the surgery. The trends and clinical implications of intracranial pressure (ICP) in these situations remain unclear. We aimed to investigate whether intracranial pressure (ICP) trends are associated with clinical outcomes in patients undergoing decompressive hemicraniectomy for MMI.

**Methods:**

This retrospective cohort study included consecutive patients with MMI who underwent decompressive craniectomy and received ICP monitoring after surgery. Using a linear mixed model, we categorized the patients into ICP increase and decrease groups based on the ICP values obtained over 192 h. We then compared the proportion of 3-month favorable outcomes (modified Rankin Scale of 0−4) and mortality rates between these groups.

**Results:**

Of 112 MMI patients who underwent decompressive hemicraniectomy, 66 (58.9%) received invasive ICP monitoring. ICP monitoring was performed for a median of 146.5 h (IQR 72.5–181.8). Among the 66 patients, 37 (56.1%) were in the ICP increase group, and 29 (43.9%) were in the ICP decrease group. During the monitoring period, the initial monitored ICP and peak ICP did not significantly differ between the ICP increase and decrease groups. However, the ICP trend was significantly different between the two groups (*P* < 0.001). In multivariable logistic regression analyses, the ICP increase group had a significantly lower proportion of 3-month favorable outcomes compared to the ICP decrease group (adjusted OR 0.11; 95% CI, 0.01–0.59; *P* = 0.019), and significantly higher mortality in the intensive care unit (adjusted OR 6.98; 95% CI, 1.37–54.6; *P* = 0.031).

**Conclusions:**

In MMI patients, continuous ICP monitoring could be useful for detecting those with an increasing ICP trend that may be associated with unfavorable clinical outcomes.

**Supplementary Information:**

The online version contains supplementary material available at 10.1186/s13613-024-01412-0.

## Introduction

Malignant middle cerebral artery infarction (MMI) has high rates of severe disability and death [1]. Various non-operative management strategies have been proposed for MMI, but no treatment has been proven to improve prognosis from randomized clinical trials [2–6]. Since 2007, randomized clinical trials on decompressive hemicraniectomy as a treatment for MMI have begun to be published [7–12]. Subsequently, decompressive hemicraniectomy has been established as a treatment that can alter the prognosis for patients who can undergo surgery within a specific time frame. However, approximately a 20−40% mortality rate was shown following decompressive hemicraniectomy [9, 10]. These deaths were closely related to progressive cerebral edema, which led to cerebral herniation even after decompressive hemicraniectomy [13]. The trends and clinical implications of intracranial pressure (ICP) in these situations remain unclear [14, 15].

ICP monitoring, one of the monitoring strategies for MMI, is commonly used in cases of traumatic brain injury and primary hemorrhagic stroke [16]. The concept of ICP is closely related to the Monro-Kellie doctrine, which assumes a rigid and intact skull [17]. However, after decompressive hemicraniectomy in MMI, the closed cranium space is expanded, and the Monro-Kellie doctrine can no longer be applied. It is unclear whether ICP increases in response to the progression of brain edema, remains unaffected or decreases due to the removal of a large skull area. It is also unknown how the clinical outcome will be if a patient experiences an increase in ICP despite undergoing hemicraniectomy. While a single cut-off value may aid in predicting mortality after decompressive surgery [15, 18], relying solely on a single point of ICP measurement for predicting prognosis can be challenging. Accordingly, it is necessary to consider not only the analysis based on a single ICP value but also the overall trend of ICP.

Considering the results of multiple randomized clinical trials that demonstrated the efficacy of decompressive surgery [7–12], we decided to investigate the significance of ICP monitoring by analyzing the ICP patterns over time after decompressive hemicraniectomy in MMI patients and to examine how these ICP patterns are associated with clinical outcomes.

## Methods

### Study population

The Institutional Review Board of Asan Medical Center approved this study and waived the need for patient-informed consent due to the retrospective design and observational nature (approval no. 2024 − 0749). Patients’ data for this study were retrieved from the neurological intensive care unit (ICU) registry and electronic medical records of Asan Medical Center from January 2009 to February 2024. We included patients who underwent decompressive hemicraniectomy for MMI from those admitted to the ICU. The results are presented according to the Strengthening the Reporting of Observational Studies in Epidemiology (STROBE) reporting guideline [19].

### MMI and decompressive hemicraniectomy

MMI was radiologically defined as magnetic resonance imaging or computed tomography showing an infarct affecting at least 50% of the middle cerebral artery territory, with or without additional infarction in the territory of the anterior or posterior cerebral artery, or an infarct volume > 145 cm^3^ on diffusion-weighted imaging [9].

Following the protocol at our stroke center, all patients with MMI were admitted to the ICU and underwent early decompressive hemicraniectomy. The decision to perform decompressive hemicraniectomy was made collaboratively through interdisciplinary discussions among neurologists, neurosurgeons, and the patients’ family members. The surgical technique has been detailed in our previous studies [14, 20, 21]. In brief, decompressive hemicraniectomy involved removing a large bone flap and performing duroplasty at the site of the MMI. A question mark-shaped skin incision was made from the ipsilateral ear to the occiput, and a large bone window was created. The dura was opened and anchored at the craniotomy’s edge to prevent epidural bleeding, followed by extensive duroplasty with artificial dura mater. Resection of the temporalis muscle and infarcted brain tissue was not performed routinely. Patients were transferred to the ICU after surgery.

### ICP monitoring

The method for ICP monitoring has been detailed in previous studies [20–22]. Briefly, in the final step of the hemicraniectomy, an ICP probe was inserted. We intended to conduct ICP monitoring in all MMI patients who underwent decompressive hemicraniectomy. However, due to equipment shortages, administrative issues, and occasional omissions of ICP probe insertion during surgery, ICP monitoring could not be performed in all patients undergoing decompressive hemicraniectomy. Subdural, intraparenchymal, or intraventricular catheters were used for ICP monitoring, with the subdural and intraparenchymal probes inserted on the ipsilateral side of the affected hemisphere. In cases where the ICP probe was not inserted during surgery, an intraparenchymal ICP probe was inserted in the contralateral hemisphere in the ICU. ICP values were continuously measured and documented hourly. Unless contraindicated, ICP measurements were taken with the head elevated to 30 degrees. The duration of ICP measurements was determined by the neurointensivist as needed, although sometimes the probe was removed earlier than expected due to malfunction. For this study, ICP values obtained for up to a maximum of 192 h from monitoring initiation were used. Cerebral perfusion pressure (CPP) was measured concurrently with ICP. CPP was calculated as the mean arterial pressure minus ICP, with the mean arterial pressure measured using an invasive arterial line. The transducer of the arterial line was positioned at the level of the right atrium. No missing values in ICP and CPP were imputed.

### Study group

**Fig. 1:**
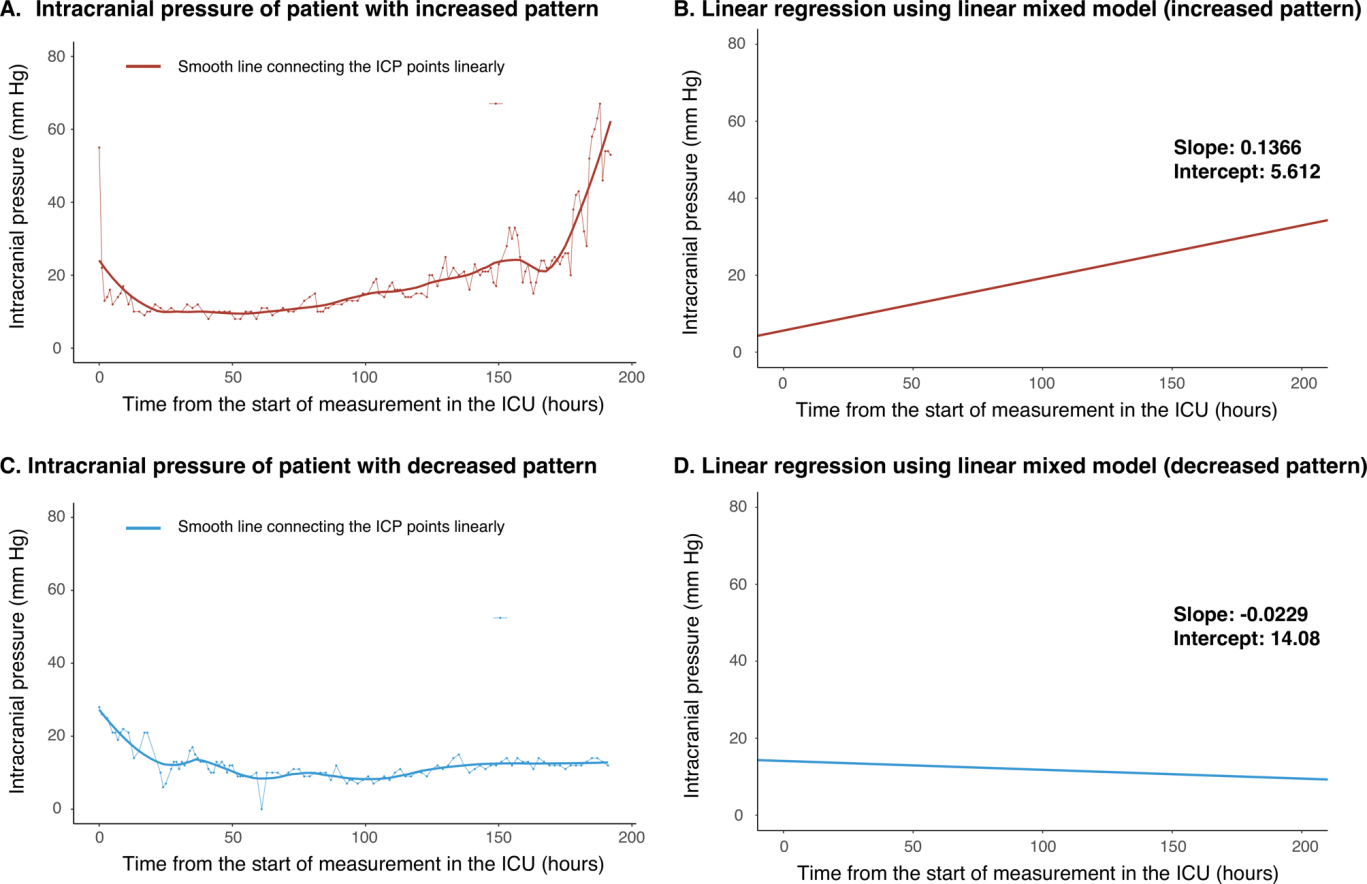
Individual trends in intracranial pressure during the monitoring period after decompressive hemicraniectomy. Fig. 1. illustrates the process of linearizing ICP values for an actual patient. **A** shows the linear connection and smooth transformation of actual ICP values from a single patient. **B** demonstrates the process of calculating the slope using a linear mixed model for the same patient depicted in **A**. **C** shows the actual ICP values of a patient from the ICP decrease group, while **D** displays the process of calculating the slope using a linear mixed model for the same patient as in **C**. Abbreviations: ICP: intracranial pressure; ICU: intensive care unit

All collected ICP data points were linearly connected over time to create individual ICP trend graphs. Using a linear mixed model, we transformed the ICP values of each patient over the observation period into a linear function and calculated the slope values (Fig. 1). Patients with a positive calculated slope value were categorized into the ICP increase (ICPi) group, while those with a negative or zero slope value were categorized into the ICP decrease (ICPd) group (Fig. 1).

### Medical management and clinical variables

Patients with MMI were managed according to guidelines from the American Heart Association/American Stroke Association, the Neurocritical Care Society, and our institutional protocol, which has been previously detailed [20, 21, 23, 24]. Briefly, rapid sequence intubation and mechanical ventilation were used for patients with impaired respiratory drive or reflexes, along with sedation and analgesia when necessary. Additional interventions included elevating the head to 30 degrees, managing fever and hyperglycemia, and administering hyperosmolar therapy with mannitol and/or hypertonic saline. For comatose patients with refractory brain swelling and intracranial hypertension despite hemicraniectomy, target temperature management and barbiturate coma therapy were employed [25, 26]. All patients underwent neurological assessments, including evaluations of pupillary light reflexes and the Glasgow Coma Scale (GCS) every hour during their ICU stay. The neurological pupillary index (NPi) was measured using an automated pupillometer starting from April 2017 (Npi-200; NeurOptics^®^, CA, USA) [27–29].

Radiological variables included the laterality of MMI, the location of the occluded vessel causing MMI, involvement of the anterior or posterior cerebral artery territory, hemorrhagic transformation of Parenchymal Hemorrhage type 2 as defined by the Second European-Australasian Acute Stroke Study (ECASS II) hemorrhage grade [30], and recanalization state using the Thrombolysis in Cerebral Infarction (TICI) scale: TICI 0 for no recanalization, TICI 1 and 2a for partial recanalization, and TICI 2b or higher for complete recanalization [31]. Time interval variables included the time from the last known well to emergency room arrival, time from emergency room arrival to skin incision, and time from the last known well to skin incision for decompressive hemicraniectomy. For in-hospital stroke or recurrence, the emergency room arrival time was replaced by the time when neurological deficits were first noted [32, 33].

### Outcomes

After discharge, patients were assessed for their functional status at 3 months through regular face-to-face visits, telephone interviews, or medical chart reviews. The primary outcome was a binary analysis of the modified Rankin Scale (mRS) score at 3 months, categorizing scores as either 0−4 for a favorable outcome or 5−6 for an unfavorable outcome. We selected a mRS of 0−4 to define a favorable outcome, aiming to reflect clinically meaningful recovery in our cohort and align with previous randomized clinical trials on decompressive hemicraniectomy in similar age groups [9, 10]. The secondary outcomes included 1-year favorable outcome, mortality in the ICU, in-hospital mortality, mortality within 3 months, mortality within 1 year, a shift analysis of the distribution of mRS scores, and length of ICU stay.

### Statistical analysis

We compared baseline characteristics based on the presence or absence of ICP monitoring. Subsequently, we compared baseline characteristics, clinical variables, management, and ICP variables according to the study groups. Continuous variables are expressed as median (IQR [interquartile range]) and categorical variables as number (percentage). The Mann-Whitney *U* test, χ² test, or Fisher’s exact test were used to compare baseline characteristics where applicable. We illustrated the ICP trend for each study group by connecting the mean values of each group over time. To compare changes in ICP and CPP over time, a random slope model from the linear mixed model was used. The changes in ICP and CPP over time for each study group were estimated using the interaction term in the random slope model, and statistical comparisons were performed.

The primary outcome was analyzed using binomial logistic regression to estimate the odds ratios (ORs) and 95% confidence intervals (CIs). Multivariable logistic regression analyses were performed to adjust for variables with *P* values smaller than 0.10 in univariable analyses. For the secondary outcomes, the common OR representing a shift in mRS scores was calculated using an ordinal logistic regression analysis. Multivariable linear regression analysis was performed for the length of ICU stay. The adjustment variables for the multivariable linear regression were the same as those for the primary outcome. Other secondary outcomes were tested using binomial logistic regression models. For the sensitivity analysis, patients exhibiting Parenchymal Hemorrhage type II on the first postoperative brain computed tomography were excluded, and the outcomes were subsequently tested [34]. A two-sided *P* < 0.05 was considered statistically significant. All data were analyzed using R version 4.2.2 (R Foundation for Statistical Computing, Vienna, Austria).

## Results

Between January 2009 and February 2024, 5,235 patients were admitted to the ICU. Out of them, 2,472 were diagnosed with ischemic stroke, and 112 stroke patients underwent decompressive hemicraniectomy for MMI. Among the MMI patients who underwent decompressive hemicraniectomy, 66 received invasive ICP monitoring. Compared with patients who did not undergo ICP monitoring, those who did had a lower incidence of smoking and more frequent anterior cerebral artery territory infarction (Supplemental Table 1).

Among the 66 patients who underwent ICP monitoring, the median age was 65.5 years (IQR, 54.8−73.0) and 32 (48.5%) were men. No significant differences were observed in demographics, medical conditions, radiologic status, and time intervals between the ICPd and ICPi groups (Table 1). Out of them, 56 (84.8%) had an ICP monitoring probe inserted during surgery. Six patients (9.1%) had an intraventricular ICP probe, all of whom had undergone a hemorrhagic transformation of Parenchymal Hemorrhage type II. The remaining 10 patients (15.2%) underwent ICP probe insertion into the contralateral hemisphere in the ICU shortly after the surgery.

**Table 1:** Baseline characteristics

	Total (*n* = 66)	ICP decrease (*n* = 29)	ICP increase (*n* = 37)	*P* value
**Demographics and medical condition**				
Age (years), median (IQR)	65.5 (54.8, 73.0)	62.0 (50.0, 72.0)	67.0 (57.0, 73.0)	0.359
Male sex, n (%)	32 (48.5)	15 (51.7)	17 (45.9)	0.641
Hypertension	37 (56.1)	17 (58.6)	20 (54.1)	0.711
Diabetes	12 (18.2)	5 (17.2)	7 (18.9)	0.861
Hyperlipidemia	14 (21.2)	8 (27.6)	6 (16.2)	0.262
Atrial fibrillation	35 (53.0)	14 (48.3)	21 (56.8)	0.493
Previous stroke history	14 (21.2)	6 (20.7)	8 (21.6)	0.927
Cancer	9 (13.6)	2 (6.9)	7 (18.9)	0.279
Smoking	10 (15.2)	2 (6.9)	8 (21.6)	0.166
IV-tPA	22 (33.3)	8 (27.6)	14 (37.8)	0.381
Endovascular thrombectomy	18 (27.3)	7 (24.1)	11 (29.7)	0.613
NIHSS at ICU admission, median (IQR)	17.5 (15.0, 20.0)	17.0 (15.0, 21.0)	18.0 (15.0, 19.0)	0.542
GCS score at ICU admission	10.0 (7.0, 13.0)	9.0 (7.0, 11.0)	12.0 (7.0, 14.0)	0.100
Premorbid mRS, median (IQR)	0.0 (0.0, 0.0)	0.0 (0.0, 0.0)	0.0 (0.0, 0.0)	0.518
**Radiologic variables**				
Laterality of infarction (right)	39 (59.1)	14 (48.3)	25 (67.6)	0.114
Occluded vessel				0.163
MCA	26 (39.4)	11 (37.9)	15 (40.5)	
Distal ICA	23 (34.8)	11 (37.9)	12 (32.4)	
Proximal ICA	14 (21.2)	4 (13.8)	10 (27.0)	
Tandem occlusion	3 (4.5)	3 (10.3)	0 (0.0)	
ACA territory involvement	41 (62.1)	19 (65.5)	22 (59.5)	0.615
PCA territory involvement	6 (9.1)	4 (13.8)	3 (8.1)	0.690
Hemorrhagic transformation	13 (19.7)	3 (10.3)	10 (27.0)	0.091
Recanalization state				0.750
No recanalization	45 (68.2)	19 (65.5)	26 (70.3)	
Partial recanalization	10 (15.2)	4 (13.8)	6 (16.2)	
Complete recanalization	11 (16.7)	6 (20.7)	5 (13.5)	
**Time intervals**				
Onset to ER time (hours), median (IQR)	5.7 (1.5, 14.7)	5.8 (1.1, 13.7)	5.7 (2.3, 14.7)	0.786
ER to decompressive surgery time (hours), median (IQR)	24.6 (19.2, 43.0)	24.8 (19.3, 37.2)	24.5 (18.7, 46.1)	0.681
Onset to decompressive surgery time (hours), median (IQR)	38.5 (24.0, 52.4)	38.5 (24.5, 44.2)	40.0 (24.0, 63.8)	0.450

There are no missing values in the baseline characteristics

Abbreviations: ACA: anterior cerebral artery; ER: emergency room; GCS: Glasgow Coma Scale; ICA: internal carotid artery; ICP: intracranial pressure; IQR: interquartile range; IV-tPA: intravenous tissue plasminogen activator; MCA: middle cerebral artery; mRS: modified Rankin Scale; NIHSS: National Institutes of Health Stroke Scale; PCA: posterior cerebral artery

### ICP trends

We evaluated 7,217 time-stamped ICP recordings. A median of 101.5 (IQR, 59.0−161.5) recordings were performed per patient during the ICP monitoring period. The median value of the first monitored ICP was 9.0 mm Hg (IQR, 4.3−13.8) and the median peak ICP during the monitoring period was 21.0 mm Hg (IQR, 14.3−27.0). The ICPi group showed significantly higher mean ICP values during the monitoring period compared with the ICPd group (ICPi, 12.2 mm Hg [IQR, 8.5−18.7] vs. ICPd, 10.3 mm Hg [IQR, 4.9−12.1]; *P* = 0.019). During the monitoring period, the initial ICP and peak ICP did not show significant differences between the ICPi and ICPd groups. Regarding complications related to ICP monitoring, there were no occurrences of ICP monitoring-related infection or hemorrhages in the study population. Device malfunction occurred in 6 patients (9.1%), with no significant difference between the ICPi group and the ICPd group (*P* = 0.392) (Table 2).

**Table 2:** Neurological and intracranial pressure-related variables

	Total (*n* = 66)	ICP decrease (*n* = 29)	ICP increase (*n* = 37)	*P* value
**Neurologic values just before decompressive hemicraniectomy**,** n (%)**				
Pupil light reflex, relevant, n (%)	45 (68.2)	18 (62.1)	27 (73.0)	0.345
Pupil light reflex, non-relevant	56 (84.8)	24 (82.8)	32 (86.5)	0.738
NPi, relevant, median (IQR)^a^ (*n* = 40)	3.2 (0.0, 4.3)	3.9 (0.0, 4.4)	3.0 (0.0, 3.9)	0.489
NPi, non-relevant^a^ (*n* = 40)	3.6 (3.3, 4.2)	3.9 (3.3, 4.4)	3.5 (3.3, 4.2)	0.399
GCS score immediately before surgery	7.0 (6.0, 10.0)	7.0 (6.0, 9.0)	7.0 (6.0, 10.0)	0.849
**Location of ICP probe**,** n (%)**				0.848
Subdural	48 (72.7)	20 (69.0)	28 (75.7)	
Intraparenchymal	12 (18.2)	6 (20.7)	6 (16.2)	
Intraventricular	6 (9.1)	3 (10.3)	3 (8.1)	
**ICP monitoring related complication**,** n (%)**				
Infection	0 (0.0)	0 (0.0)	0 (0.0)	-
Hemorrhage	0 (0.0)	0 (0.0)	0 (0.0)	-
Device malfunction	6 (9.1)	4 (13.8)	2 (5.4)	0.392
**After decompressive hemicraniectomy (ICP)**,** median (IQR)**				
Duration of ICP monitoring, hours	146.5 (72.5, 181.8)	148.0 (82.0, 173.0)	145.0 (72.0, 183.0)	0.712
Numbers of ICP measurement	101.5 (59.0, 161.5)	115.0 (58.0, 159.0)	101.0 (63.0, 171.0)	> 0.999
First monitored ICP, mm Hg	9.0 (4.3, 13.8)	10.0 (4.0, 16.0)	8.0 (5.0, 13.0)	0.641
Peak ICP during ICP, mm Hg monitoring, mm Hg	21.0 (14.3, 27.0)	18.0 (13.0, 25.0)	21.0 (15.0, 34.0)	0.149
Mean ICP during ICP monitoring, mm Hg	10.5 (6.6, 14.8)	10.3 (4.9, 12.1)	12.2 (8.5, 18.7)	**0.019**
**After decompressive hemicraniectomy (CPP)**,** median (IQR)**				
Duration of CPP monitoring, hours	142.0 (72.0, 175.0)	142.0 (76.0, 167.0)	143.0 (63.8, 177.8)	0.911
Numbers of CPP measurement	97.0 (53.0, 158.0)	98.0 (54.0, 156.0)	89.0 (50.5, 160.3)	0.942
First monitored CPP, mm Hg	81.0 (69.0, 95.0)	81.0 (67.0, 92.0)	80.5 (70.8, 95.3)	0.731
The lowest CPP during CPP monitoring, mm Hg	52.0 (42.0, 60.0)	54.0 (49.0, 60.0)	45.0 (33.8, 59.8)	0.053
Mean CPP during CPP monitoring, mm Hg	76.4 (69.7, 81.5)	76.9 (73.0, 81.9)	75.4 (65.5, 81.4)	0.065

Abbreviations: CPP: cerebral perfusion pressure; GCS: Glasgow Coma Scale; ICP: intracranial pressure; IQR: interquartile range; NPi: Neurological Pupil index

^a^ Only patients after April 1, 2017, were measured for NPi

**Fig. 2:**
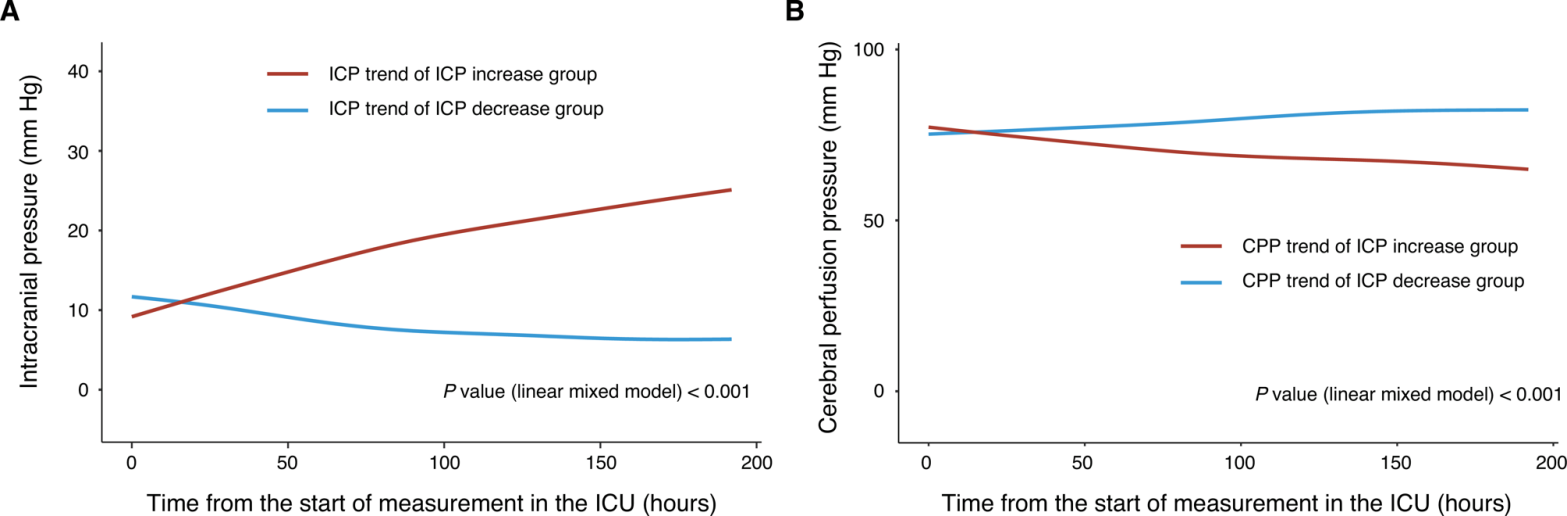
Trends of intracranial pressure and cerebral perfusion pressure according to the study groups. **A** shows the ICP trend in the ICP increase and decrease groups. Using a linear mixed model, ICP increase group. The ICP increase group exhibited a median daily ICP rise of 1.0 mm Hg (IQR, 0.4−8.3), while the ICP decrease group showed a median daily decrease of 0.9 mm Hg (IQR, 0.4−1.4). **B** illustrates the CPP trends according to the study groups. The ICP increase group showed a median daily decrease in CPP of 1.6 mm Hg (IQR, -0.6−9.9), and the ICP decrease group showed a median daily increase of 1.6 mm Hg (IQR, 0.4−2.5). Abbreviations: CPP: cerebral perfusion pressure; ICP: intracranial pressure; IQR: interquartile range

To analyze the trend of ICP for individuals within the groups, we depicted individual ICP curves (Supplemental Fig. 1). In the linear mixed model analysis, the decline of ICP per day was 0.9 mm Hg (IQR, 0.4–1.4) in the ICPd group. In the ICPi group, the daily increase in ICP was 1.0 mm Hg (IQR, 0.4–8.3) (Fig. 2A). The slope was significantly different between the ICPd and ICPi groups (linear mixed model; *P* < 0.001, Fig. 2A). In addition, the CPP in the ICPi group showed a decreasing trend, while the CPP in the ICPd group exhibited an increasing trend. Significant differences in CPP trends were observed between the study groups (linear mixed model; *P* < 0.001, Fig. 2B). There were no significant differences in the management of increased ICP between the study groups (Supplemental Table 2).

### Clinical outcomes

**Fig. 3:**
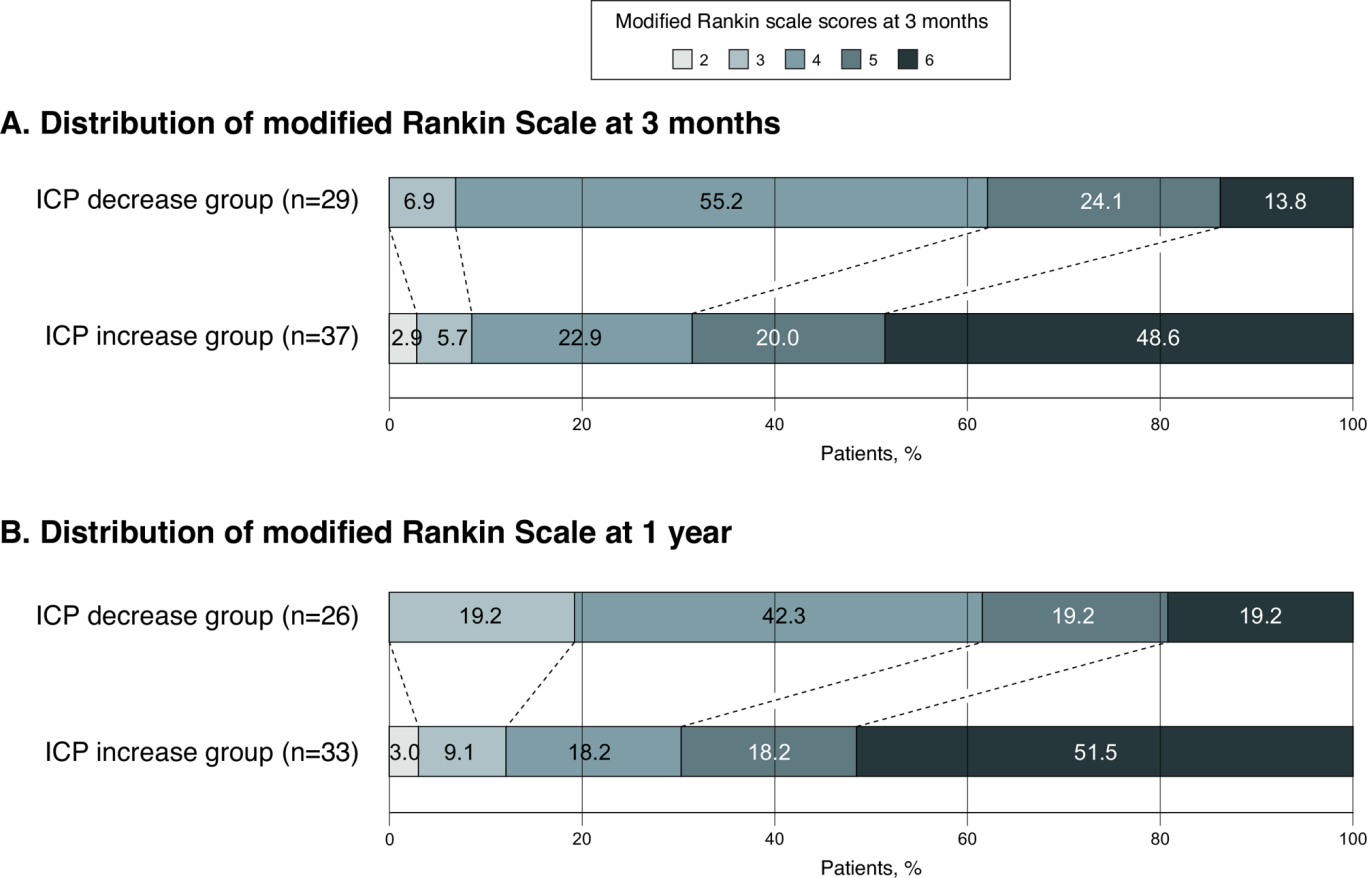
Distribution of modified Rankin Scale scores at 3 months and at 1 year according to the study groups. The median mRS score in the ICP decrease group was 4 (IQR, 4–5) at both 3 months and 1 year, while in the ICP increase group, it was 5 (IQR, 4–6) at 3 months and 6 (IQR, 4–6) at 1 year. Abbreviations: ICP: intracranial pressure; mRS: modified Rankin Scale

In the unadjusted model, a significantly lower proportion of patients in the ICPi group achieved a 3-month favorable outcome compared with those in the ICPd group (OR, 0.26; 95% CI, 0.09−0.71; *P* = 0.010). On the multivariable analysis, statistical significance between the two groups was maintained in the 3-month favorable outcome (29.7% vs. 62.1%; OR, 0.11; 95% CI, 0.01−0.59; *P* = 0.019, Table 3; Fig. 3).

**Table 3:** Clinical outcomes

	ICP decrease (*n* = 29)^a^	ICP increase (*n* = 37)^a^	Unadjusted OR (95% CI)	Adjusted OR (95% CI)	*P* value
**Primary outcome**					
3-month favorable outcome (mRS of 0–4)^b^	18/29 (62.1)	11/37 (29.7)	0.26 (0.09, 0.71)	0.11 (0.01, 0.59)	**0.019**
**Secondary outcomes**					
1-year favorable outcome (mRS of 0–4)^b^ (*n* = 59)	16/26 (61.5)	10/33 (30.3)	0.27 (0.09, 0.79)	0.11 (0.01, 0.62)	**0.023**
Mortality in the ICU^c^	2/29 (6.9)	15/37 (40.5)	9.20 (2.28, 62.5)	6.98 (1.37, 54.6)	**0.031**
In-hospital mortality^c^	3/29 (10.3)	15/37 (40.5)	5.91 (1.68, 28.0)	3.81 (0.88, 20.4)	0.086
Mortality within 3 months^c^ (*n* = 64)	4/29 (13.8)	17/35 (48.6)	5.90 (1.83, 23.3)	4.74 (0.99, 23.6)	0.061
Mortality within 1 year^c^ (*n* = 59)	5/26 (18.2)	17/33 (51.5)	4.46 (1.43, 16.0)	2.68 (0.65, 12.0)	0.177
Shift of mRS score at 3 months^d^ (*n* = 64)	4.0 (4.0, 5.0)	5.0 (4.0, 6.0)	3.24 (1.28, 8.47)	2.41 (0.80, 7.60)	0.122
Length of ICU stay^e^	17.0 (12.0, 22.0)	13.0 (10.0, 21.0)	-1.55 (-5.75, 2.65)	-0.38 (-5.62, 3.31)	0.614

Abbreviations: ICP: intracranial pressure; ICU: neurological intensive care unit; mRS: modified Rankin Scale; OR: odds ratio

^a^ Data are presented as the number (percentage) of patients for categorical variables and median (IQR) for ordinal variables

^b^ Treatment effects are analyzed with binary logistic regression adjusted for variables of age, hypertension, previous stroke history, anterior cerebral artery territory involvement, and mean ICP during the monitoring period

^c^ Treatment effects are analyzed with binary logistic regression adjusted for variables of age, hypertension, hemorrhagic transformation, and mean ICP during the monitoring period

^d^ Treatment effects are analyzed with ordinal logistic regression adjusted for variables of age, hypertension, previous stroke history, anterior cerebral artery territory involvement, and mean ICP during the monitoring period

^e^ Treatment effects are analyzed with multivariable linear regression adjusted for variables of age, hypertension, previous stroke history, anterior cerebral artery territory involvement, and mean ICP during the monitoring period

Seventeen patients (25.8%) died in the ICU, with 21 (32.8%) mortality within 3 months and 22 (37.3%) within 1 year. Of the 22 patients who died within 1 year, 20 (90.9%) died due to neurological causes. Of the remaining two patients (9.1%), one died from septic shock and the other from mesenteric ischemia. Four patients underwent withdrawal of life-sustaining treatments, and two received withholding of life-sustaining treatments; all of these six patients died in the hospital.

**Fig. 4:**
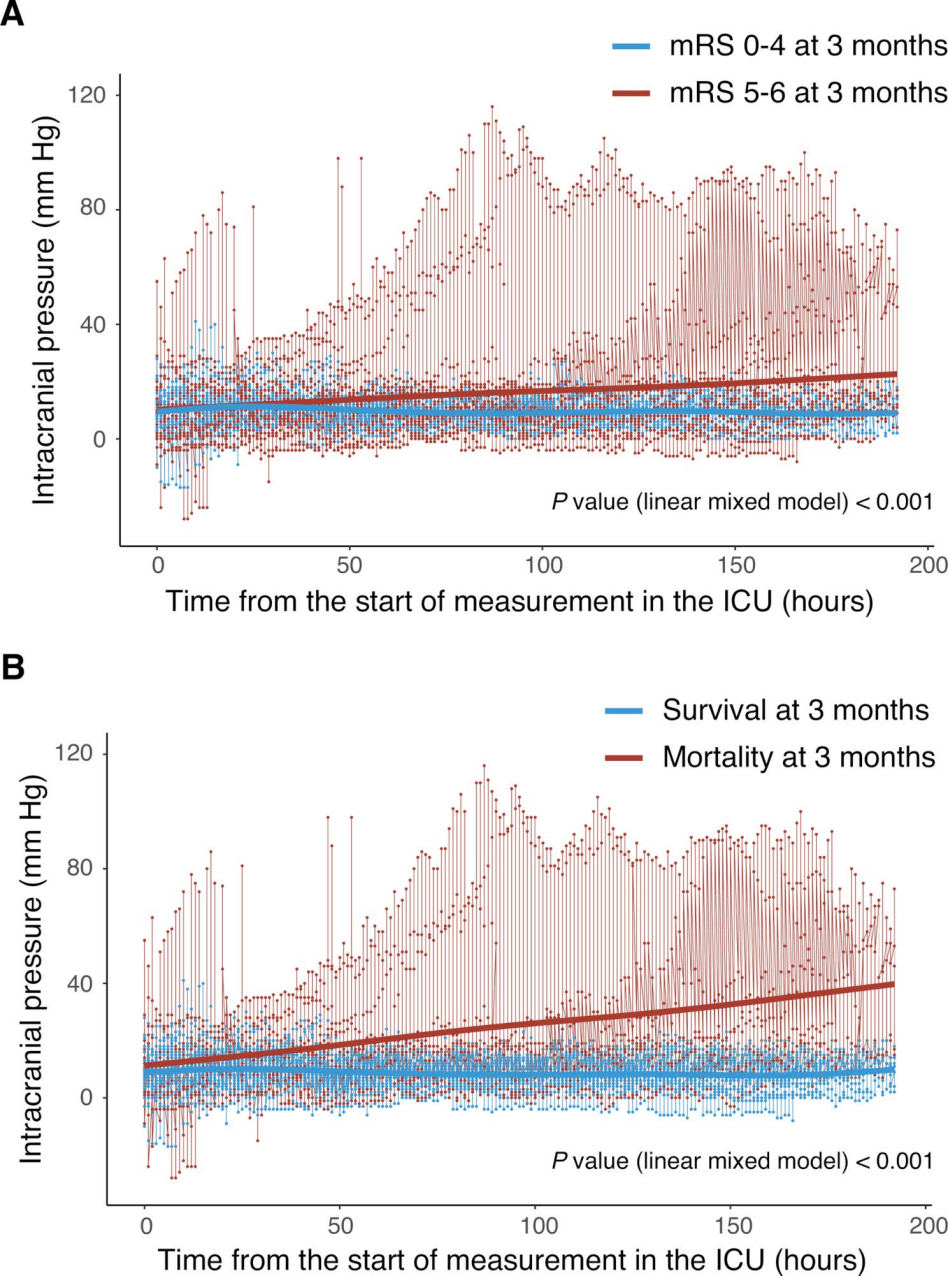
Trends of intracranial pressure according to the outcomes. **A** shows the ICP trend according to 3-month mRS scores of 0–4 and 5–6, connecting the median ICP values at each time point. **B** illustrates the ICP trend based on 3-month survival and mortality, again connecting the median ICP values at each time point. Abbreviations: ICP: intracranial pressure; ICU: intensive care unit; mRS: modified Rankin Scale

The ICPi group had a lower 1-year favorable outcome (OR, 0.11; 95% CI, 0.01−0.62; *P* = 0.023) and higher ICU mortality (OR, 6.98; 95% CI, 1.37−54.6; *P* = 0.031) compared with the ICPd group. Regarding the other secondary outcomes, there were no significant differences in the mRS shift analysis, in-hospital mortality, mortality within 3 months, mortality within 1 year, or length of ICU stay between the study groups (Table 3). Additionally, we analyzed ICP trends according to the primary outcome and mortality within 3 months. Patients with a 3-month unfavorable outcome showed an increasing ICP trend compared with those with a 3-month favorable outcome (linear mixed model; *P* < 0.001). Similarly, patients who died within 3 months also exhibited an increasing ICP trend compared with those who survived (linear mixed model; *P* < 0.001) (Fig. 4 and Supplemental Fig. 2).

### Sensitivity analysis

Since massive intracerebral hemorrhage significantly impacts prognosis, a sensitivity analysis was performed on MMI patients without hemorrhagic transformation of Parenchymal Hemorrhage type II. When excluding patients with Parenchymal Hemorrhage type II, the proportion of survival without severe disability at 3 months showed a significant difference between the study groups, consistent with previous results (65.4% vs. 33.3%; OR, 0.11; 95% CI, 0.01−0.74; *P* = 0.042, Supplemental Table 3). There were no statistically significant differences between groups in the mRS shift analysis, in-hospital mortality, mortality within 3 months, or length of ICU stay (Supplemental Table 3).

## Discussion

In this cohort study, we found that MMI patients who underwent decompressive hemicraniectomy and showed an increasing ICP pattern had approximately 9 times higher odds of an unfavorable outcome at 3 months and 1 year, and approximately 7 times higher odds of ICU mortality compared with those who showed a decreasing ICP pattern. Importantly, we delineated how ICP changes over time following decompressive hemicraniectomy, once the rigid skull is no longer present. These trends in ICP, obtained through serial monitoring, could be associated with clinical outcomes. These findings indicate that ICP monitoring may help identify patients with an increasing ICP trend despite undergoing decompressive hemicraniectomy, who were shown to have higher mortality rates and a lower likelihood of achieving favorable neurological outcomes.

The ICP dynamics are governed by the Monro-Kellie doctrine, which posits that the volume of intracranial contents remains constant within the closed cranial vault [35, 36]. When a decompressive hemicraniectomy is conducted, the dynamics of ICP are modified due to the removal of a section of the skull. After a hemicraniectomy, the rigid constraints of the skull are removed, allowing the brain to expand outward through the surgical opening, which can relieve elevated ICP caused by MMI. However, because the cranium is no longer a completely closed system after the hemicraniectomy, the premise of the Monro-Kellie doctrine is no longer valid. The brain’s capacity to expand outward could alter the relationship between intracranial volume and ICP.

Even if the Monro-Kellie doctrine does not strictly apply, ICP can still increase due to the presence of the reconstructed dura mater and scalp instead of bone [37]. This increase in ICP may occur as the dura mater and scalp tissue reach their expansion limits and are gradually compressed by the expanding infarcted brain tissue. Therefore, monitoring the dynamic changes in brain tissue within the scalp and dura mater through ICP monitoring could be clinically beneficial.

Previous studies only measured ICP immediately after surgery or in the early stages, failing to provide data on the progression of cerebral edema over time. By conducting long-term ICP monitoring and observing trends, we were able to identify the relationship between ICP trends and clinical outcomes. In addition, the baseline ICP values measured in patients with a portion of the skull removed may not be accurate due to the inability to fully apply the Monro-Kellie doctrine. After decompressive hemicraniectomy, the cranium is no longer closed, which can lead to a wide range of ICP values. Therefore, monitoring the trend of ICP rather than relying on a single measurement may be more reasonable in the post-craniectomy state.

ICP monitoring could benefit MMI patients who have undergone decompressive hemicraniectomy by detecting increases in ICP and enabling appropriate treatment interventions. However, there were considerable debates regarding whether treatment decisions based on ICP monitoring affected outcomes in MMI patients who underwent decompressive hemicraniectomy, and whether ICP values were associated with prognosis. Several studies have shown that ICP monitoring in patients with MMI was inappropriate for reflecting the status of cerebral edema [38–40]. In addition, there was no evidence that treatment based on ICP monitoring improves prognosis [40]. Conversely, other studies have suggested that specific ICP values in patients with MMI after decompressive hemicraniectomy are associated with cerebral edema and prognosis [14, 15, 18, 41]. Considering these controversial findings, we conducted an analysis to verify the relationship between ICP trends and prognosis, using ICP values obtained hourly for up to 8 days. ICP is closely associated with CPP, and an increase in ICP can lead to a decrease in CPP. Specifically, a sudden increase in ICP affects CPP reduction, potentially causing ischemia in the penumbra area where hypoperfusion persists [42]. This additional ischemia could lead to a larger infarction volume, further mass effect, and edematous changes, creating a vicious cycle that results in a poor prognosis. Since transient increases in ICP at specific time points may not fully explain sustained decreases in CPP, observing trends over a sufficient period through continuous ICP monitoring could potentially reveal CPP trends. In our study, although there were no statistical differences between the ICPd group and the ICPi group in terms of the first monitored ICP and peak ICP values, significant variances were observed in the mean ICP and the trend of ICP over time throughout the monitoring period. These results suggest that specific ICP values and ICP trends might reflect different aspects of the patient’s condition. Thus, not only the instantaneous values, such as peak and first monitored ICP, but also the trends in ICP were related to clinical outcomes, emphasizing the importance of paying closer attention to these trends. Ultimately, continuous ICP monitoring could detect a decreasing CPP trend, and this declining CPP trend could be associated with additional ischemia, leading to poor clinical outcomes.

In MMI, cerebral edema initially worsens following the onset of infarction but generally shows improvement after a specific time point (median 6 days; IQR 4−7) [21, 43–45]. After a specific time point, many patients with MMI experience a reduced degree of herniation and show no further neurological deterioration, indicating that management during the acute phase of the first week might be even more crucial than in other types of acute brain injury [46]. Although there is no Class I evidence, extensive efforts are made to manage the acute phase of MMI. We found that our study did not reach statistical significance for three-month mortality or in-hospital mortality; however, the mortality rate within the ICU was significantly higher in the ICPi group compared with the ICPd group. The fact that mortality within the ICU represents an acute phase suggests that an increasing ICP trend following decompressive hemicraniectomy may reflect the worsening of acute cerebral edema.

The beneficial effect of inserting an invasive ICP monitoring probe without hemicraniectomy in MMI is unclear. However, simultaneous insertion during decompressive hemicraniectomy presents minimal technical challenges. Additionally, our results showed no complications such as infection or major bleeding from ICP probe insertion. Given the absence of clear treatment guidelines following decompressive hemicraniectomy for MMI, considering ICP monitoring for patients undergoing decompressive hemicraniectomy may assist in decision-making for treatment, as the risks associated with probe insertion were minimal, and it could be carried out simultaneously with the surgery. While decompressive hemicraniectomy could improve functional outcomes for specific patient groups, its primary objective is as a life-saving treatment. The increase in ICP following decompressive hemicraniectomy was associated with mortality during the ICU stay, which can help guide caregivers in understanding the patient’s prognosis. Furthermore, this increasing trend in ICP may indicate the need for further brain imaging to assess the requirement for additional treatments.

In addition to the inherent limitations of a single-center, retrospective design, our study has other limitations of note. First, approximately 40% of patients did not receive ICP monitoring after decompressive hemicraniectomy. Due to the retrospective nature of the study, the reasons for not conducting monitoring were not documented, raising potential concerns about selection bias. Nonetheless, there were no statistically significant differences in the majority of baseline characteristics between the patients who underwent ICP monitoring and those who did not. Second, our study did not include monitoring or analysis of brain tissue oxygenation [47]. Consequently, we could not consider the impact of brain hypoxia burden on treatment effectiveness. However, this limitation arises from the unavailability of brain tissue oxygenation, which was previously utilized in the country where the study was conducted. Third, the lack of blinding in ICP monitoring raises the possibility of a self-fulfilling prophecy. Being aware of increasing ICP trends, clinicians may have adjusted the intensity of interventions or considered withdrawing life-sustaining treatments. Although these clinical decisions were based on established protocols and ethical guidelines, knowledge of ICP status could still affect clinicians’ judgment. Future studies could mitigate this potential bias by incorporating a blinded or partially blinded approach to ICP monitoring. Finally, the clinical outcomes of included patients are not solely determined by the presence of ICP monitoring but also by the administration of corresponding treatments. Moreover, poor outcomes may arise from various factors, including ICP status and comorbid conditions. Although the association between ICP trends and clinical outcomes persists even after multivariable adjustments, there remains the limitation of potential unadjusted confounders.

## Conclusion

We found that ICP monitoring helps identify patients with a trend of increasing ICP, which indicates inadequate decompression due to the progression of brain edema, despite undergoing decompressive hemicraniectomy for MMI. Such an increase in the ICP trend was associated with higher ICU mortality rates and unfavorable neurological outcomes.

## Electronic supplementary material

Below is the link to the electronic supplementary material.

Supplementary Material 1

Supplementary Material 2

## Data Availability

The anonymized dataset will be available on reasonable request to the corresponding author.

## Abbreviations

CI: Confidence interval
CPP: Cerebral perfusion pressure
ECASS II: Second European-Australasian Acute Stroke Study
GCS: Glasgow Coma Scale
ICP: Intracranial pressure
ICPd: Intracranial pressure decrease
ICPi: Intracranial pressure increase
ICU: Intensive care unit
IQR: Interquartile range
MMI: Malignant middle cerebral artery infarction
mRS: Modified Rankin Scale
NPi: Neurological pupillary index
OR: Odds ratio
TICI: Thrombolysis in Cerebral Infarction
