# Supplementary material for: Intracranial pressure trends and clinical outcomes after decompressive hemicraniectomy in malignant middle cerebral artery infarction
Source: Ann Intensive Care. 2024 Nov 27;14:176. doi: 10.1186/s13613-024-01412-0 (PMC11599697; doi:10.1186/s13613-024-01412-0)
Supplement: Supplementary file 2 — Supplementary Material 2 [file 13613_2024_1412_MOESM2_ESM.docx]

**Supplemental Materials**

**Intracranial Pressure Trends and Clinical Outcomes After Decompressive Hemicraniectomy in Malignant Middle Cerebral Artery Infarction**

**Supplemental Fig. 1**. Each patient's intracranial pressure trend according to the study groups

**Supplemental Fig. 2**. Each patient's intracranial pressure trend according to the outcomes

# Supplemental Table 1. Baseline characteristics according to the presence of intracranial pressure monitoring

# Supplemental Table 2. Management for increased intracranial pressure

**Supplemental Table 3.** Clinical outcomes of patients without hemorrhagic transformation

**Supplemental Fig. 1**. Each patient's intracranial pressure trend according to the study groups


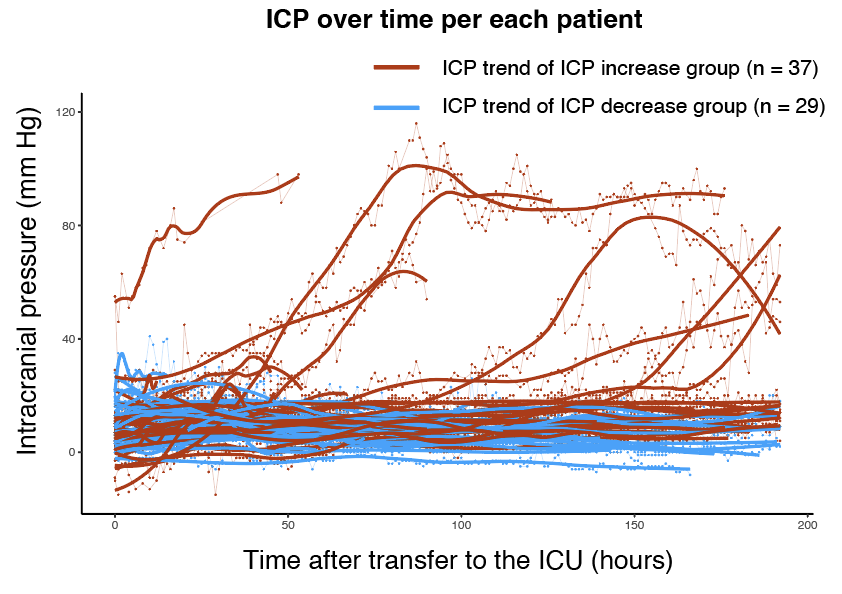


The ICP trends for each patient were transformed into smoothed lines. Patients in the ICP increase group are indicated with red lines, while patients in the ICP decrease group are indicated with blue lines.

Abbreviations: ICP, intracranial pressure; ICU, intensive care unit.

**Supplemental Fig. 2**. Each patient's intracranial pressure trend according to the outcomes


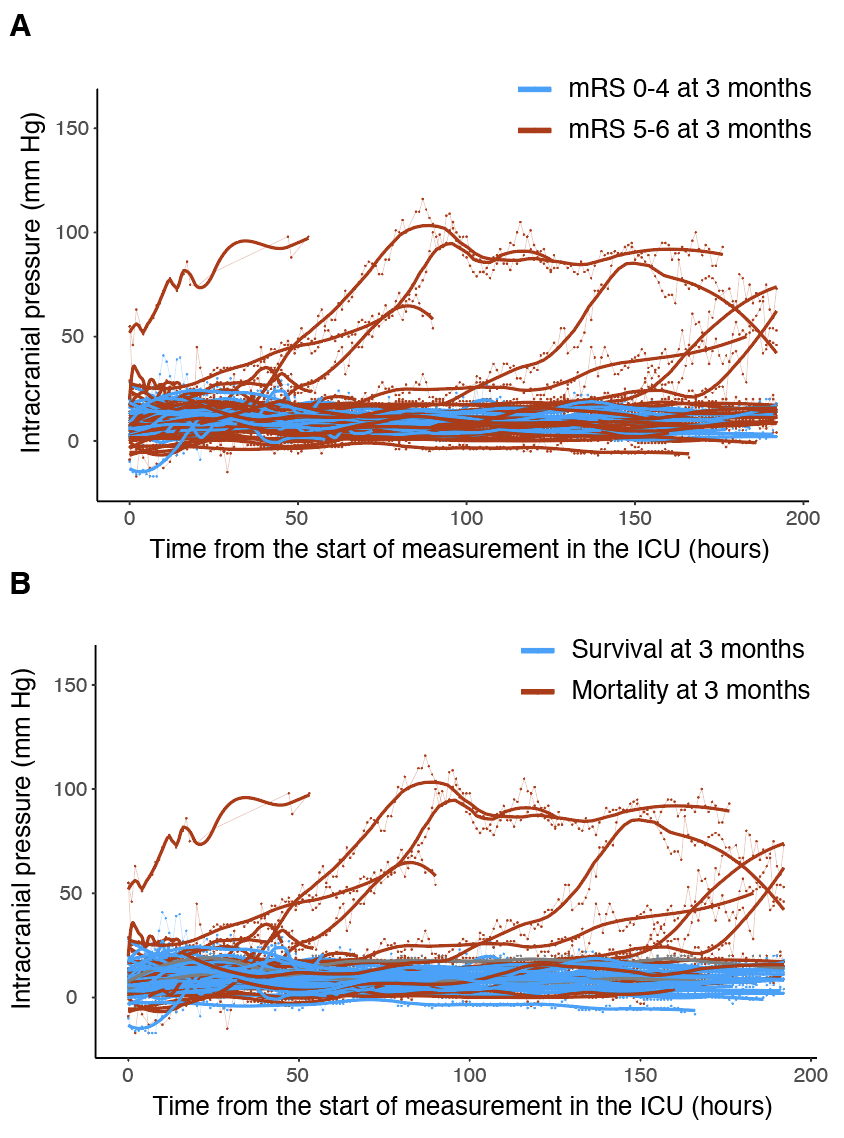


The ICP trends for each patient are transformed into smoothed lines. In Fig. S2A, the 3-month mRS 0-4 group is indicated in blue, while the 3-month mRS 5-6 group is indicated in red. In Fig. S2B, 3-month survivors are shown in blue, and 3-month non-survivors are shown in red.

Abbreviations: ICP, intracranial pressure; ICU, intensive care unit; mRS, modified Rankin Scale.

# Supplemental Table 1. Baseline characteristics according to the presence of intracranial pressure monitoring

|  | Total (n = 112) | ICP monitoring (+) (n = 66) | ICP monitoring (-) (n = 46) | *P* value |
| --- | --- | --- | --- | --- |
| **Demographics and medical condition** |  |  |  |  |
| Age (years), median (IQR) | 65.0 (58.0, 73.0) | 65.5 (54.8, 73.0) | 65.0 (60.3, 72.9) | 0.435 |
| Male sex, n (%) | 57 (50.9) | 32 (48.5) | 25 (54.3) | 0.541 |
| Hypertension | 66 (58.9) | 37 (56.1) | 29 (63.0) | 0.460 |
| Diabetes | 20 (17.9) | 12 (18.2) | 8 (17.4) | 0.914 |
| Hyperlipidemia | 28 (25.0) | 14 (21.2) | 14 (30.4) | 0.267 |
| Atrial fibrillation | 60 (53.6) | 35 (53.0) | 25 (54.3) | 0.891 |
| Previous stroke history | 24 (21.4) | 14 (21.2) | 10 (21.7) | 0.947 |
| Cancer | 18 (16.1) | 9 (13.6) | 9 (19.6) | 0.401 |
| Smoking | 26 (23.2) | 10 (15.2) | 16 (34.8) | **0.015** |
| IV-tPA | 38 (33.9) | 22 (33.3) | 16 (34.8) | 0.873 |
| Endovascular thrombectomy | 27 (24.1) | 18 (27.3) | 9 (19.6) | 0.348 |
| NIHSS at ICU admission, median (IQR) | 17.0 (14.0, 20.0) | 17.5 (15.0, 20.0) | 16.5 (13.0, 19.0) | 0.276 |
| GCS score at ICU admission | 10.0 (8.0, 13.0) | 10.0 (7.0, 13.0) | 10.0 (9.0, 13.0) | 0.309 |
| Premorbid mRS, median (IQR) | 0.0 (0.0, 0.0) | 0.0 (0.0, 0.0) | 0.0 (0.0, 0.0) | 0.882 |
| **Radiologic variables** |  |  |  |  |
| Laterality of infarction (right) | 72 (64.3) | 39 (59.1) | 33 (71.7) | 0.169 |
| Occluded vessel |  |  |  | 0.551 |
| MCA | 43 (38.4) | 26 (39.4) | 17 (37.0) |  |
| Distal ICA | 35 (31.3) | 23 (34.8) | 12 (26.1) |  |
| Proximal ICA | 29 (25.9) | 14 (21.2) | 15 (32.6) |  |
| Tandem occlusion | 5 (4.5) | 3 (4.5) | 2 (4.3) |  |
| ACA territory involvement | 57 (50.9) | 41 (62.1) | 16 (34.8) | **0.004** |
| PCA territory involvement | 12 (10.7) | 7 (10.6) | 6 (13.0) | 0.692 |
| Recanalization state |  |  |  | 0.279 |
| No recanalization | 80 (71.4) | 45 (68.2) | 35 (76.1) |  |
| Partial recanalization | 18 (16.1) | 10 (15.2) | 8 (17.4) |  |
| Complete recanalization | 14 (12.5) | 11 (16.7) | 3 (6.5) |  |
| **Time intervals** |  |  |  |  |
| Onset to ER time (hours), median (IQR) | 5.9 (1.7, 14.9) | 5.7 (1.5, 14.3) | 7.9 (2.0, 16.5) | 0.377 |
| ER to decompressive surgery time (hours), median (IQR) | 24.8 (18.2, 44.1) | 24.6 (19.2, 43.0) | 24.8 (17.3, 44.4) | 0.734 |
| Onset to decompressive surgery time (hours), median (IQR) | 39.3 (23.8, 56.6) | 38.5 (24.0, 52.4) | 41.0 (22.3, 60.5) | 0.617 |

Abbreviations: ACA, anterior cerebral artery; ER, emergency room; GCS, Glasgow Coma Scale; ICA, internal carotid artery; ICP, intracranial pressure; IQR, interquartile range; IV-tPA, intravenous tissue plasminogen activator; MCA, middle cerebral artery; mRS, modified Rankin Scale; NIHSS, National Institutes of Health Stroke Scale; PCA, posterior cerebral artery.

**Supplemental Table 2**. Management for increased intracranial pressure

| **Management** | Total (n = 66) | ICP decrease (n = 29) | ICP increase (n = 37) | *P* value |
| --- | --- | --- | --- | --- |
| Mannitol therapy, n (%) | 62 (93.9) | 26 (89.7) | 36 (97.3) | 0.312 |
| Hypertonic saline therapy | 28 (42.4) | 12 (41.4) | 16 (43.2) | 0.879 |
| Barbiturate coma therapy | 16 (24.2) | 4 (13.8) | 12 (32.4) | 0.079 |
| Targeted temperature management | 31 (47.0) | 12 (41.4) | 19 (51.4) | 0.420 |
| Duration of targeted temperature management (day), median (IQR) | 7.0 (6.0, 12.0) | 7.0 (6.0, 13.0) | 7.0 (6.0, 10.8) | 0.821 |

Abbreviations: ICP, intracranial pressure; ICU, intensive care unit.

**Supplemental Table 3.** Clinical outcomes of patients without hemorrhagic transformation

|  | ICP decrease  (n = 26)^a^ | ICP increase  (n = 27)^a^ | Unadjusted OR  (95% CI) | Adjusted OR  (95% CI) | *P* value |
| --- | --- | --- | --- | --- | --- |
| **Primary outcome** |  |  |  |  |  |
| 3-month favorable outcome (mRS of 0-4)^b^ | 17/26  (65.4) | 9/27  (33.3) | 0.26  (0.08, 0.80) | 0.11  (0.01, 0.74) | **0.042** |
|  |  |  |  |  |  |
| **Secondary outcomes** |  |  |  |  |  |
| 1-year favorable outcome (mRS of 0-4)^b^ (n = 47) | 15/23  (65.2) | 8/24  (33.3) | 0.27  (0.08, 0.87) | 0.09  (0.01, 0.69) | **0.039** |
| Mortality in the ICU^c^ | 1/26  (3.8) | 10/27  (37.0) | 14.7  (2.48, 283) | 14.1  (2.02, 292) | **0.022** |
| In-hospital mortality^c^ | 2/26  (7.7) | 15/27  (37.0) | 7.06  (1.61, 49.9) | 5.75  (1.13, 44.2) | 0.051 |
| Mortality within 3 months^c^ (n = 52) | 3/26  (11.5) | 12/26  (46.2) | 6.57  (1.73, 32.7) | 5.07  (1.08, 30.0) | 0.050 |
| Mortality within 1 year^c^ (n = 47) | 3/23  (13.0) | 12/24  (50.0) | 6.67  (1.71, 33.9) | 5.42  (1.13, 32.8) | **0.044** |
| Shift of mRS score at 3 months^d^ (n = 52) | 4.0  (4.0, 5.0) | 5.0  (4.0, 6.0) | 3.33  (1.19, 9.77) | 2.28  (0.68, 8.04) | 0.189 |
| Length of ICU stay^e^ | 17.0  (12.0, 22.0) | 12.0  (10.0, 20.0) | -1.79  (-6.53, 2.695) | -1.37  (-6.35, 3.61) | 0.592 |

Abbreviations: ICP, intracranial pressure; ICU, neurological intensive care unit; mRS, modified Rankin Scale; OR, odds ratio.

^a^ Data are presented as the number (percentage) of patients for categorical variables and median (IQR) for ordinal variables.

^b^ Treatment effects are analyzed with binary logistic regression adjusted for variables of age, hypertension, previous stroke history, anterior cerebral artery territory involvement, and mean ICP during the monitoring period.

^c^ Treatment effects are analyzed with binary logistic regression adjusted for variables of age, hypertension, and mean ICP during the monitoring period.

^d^ Treatment effects are analyzed with ordinal logistic regression adjusted for variables of age, hypertension, previous stroke history, anterior cerebral artery territory involvement, and mean ICP during the monitoring period.

^e^ Treatment effects are analyzed with multivariable linear regression adjusted for variables of age, hypertension, previous stroke history, anterior cerebral artery territory involvement, and mean ICP during the monitoring period.
